# Delayed Local Recurrence Following Radiation Therapy for Muscle-Invasive Bladder Cancer Emphasizing the Need for Lifelong Surveillance: a Case Report

**DOI:** 10.1100/tsw.2008.34

**Published:** 2008-02-19

**Authors:** Rajitha Sunkara, Rajinikanth Ayyathurai, Alan Nieder, Murugesan Manoharan

**Affiliations:** Department of Urology, University of Miami Miller School of Medicine, Miami, FL, USA

**Keywords:** bladder cancer, delayed local recurrence, surveillance, muscle invasive

## Abstract

We report a case of 68-years-old gentleman who developed a delayed local recurrence, 30 years following curative radiation treatment for muscle-invasive bladder cancer. This case emphasizes the importance of lifelong post-treatment surveillance for bladder cancer.

## INTRODUCTION

Bladder cancer is the fourth most common malignancy in men in the U.S. In particular, muscle-invasive bladder cancer has a high mortality rate[1].The objective of this case is to emphasize the need for lifelong surveillance for bladder cancer.

## CASE REPORT

A 68-year-old gentleman presented with a 6-week history of dysuria and hematuria. He was neither a smoker nor had any occupational exposure for bladder cancer. Thirty years ago (1978), the patient was diagnosed with stage T1, high-grade, urothelial cancer of the bladder. Initially, he was treated with intravesical BCG. Three years later, he developed tumor recurrence. Subsequent transurethral resection of the bladder tumor revealed muscle-invasive cancer.

In 1981, he was treated with external beam radiation therapy (EBRT) at Memorial Sloan Kettering Cancer Center, New York. The treatment schedule and precise dosage of the radiation therapy is not available. He was initially followed with cytology and cystoscopy every 6 months during the first 5 years, and subsequently with yearly cystoscopy and cytology for the last 25 years. No disease recurrence was noted.

The most recent cystoscopy performed in March 2007 demonstrated a 3-cm nodular lesion at the trigone. He underwent a transurethral resection of the lesion. The pathology demonstrated a high-grade, muscle-invasive, urothelial cancer. Subsequently, the patient was referred to us for further management. His metastatic workup with CT scan of the abdomen and pelvis and bone scan were negative.

The patient underwent a cystoprostatectomy with ileal conduit creation in April 2007. The pathology revealed a 2.5-cm, infiltrating, high-grade, urothelial carcinoma extending into the perivesical adipose tissue (pT3a) .There was no evidence of perineural or lymphovascular invasion and the margins were negative. Extensive mucosal ulceration with stromal reactive changes and transitional cell carcinoma *in situ* were also observed. His postoperative course was uneventful and he was discharged home on the 7$^{th}$ postoperative day. He is currently receiving adjuvant chemotherapy with Cisplatin and Gemcitabine.

## COMMENTS

Urothelial cancer is uncommon before the age of 40 and represents less than 4% of all bladder tumors[2]. In our case, this gentleman initially presented with muscle-invasive bladder cancer at a relatively young age of 38 years. The treatment of bladder cancer is based on the grade and stage of the tumor irrespective of the age of the patient[3]. The treatment modalities for muscle-invasive bladder cancer include cystectomy, EBRT, chemotherapy, and combined modality approaches[3].

**Table 1**
**Local relapse-free rates for patients with T 2-T4 disease following radiation therapy.**

| Local relapse- free rate | T2 (%) | T3 (%) | T4 (%) | Overall (%) |
|---|---|---|---|---|
| 5-yr | 44.5 | 27 | 0 | 34 |
| 10-yr | 44.5 | 26 | 0 | 32 |

This patient received curative radical EBRT, which is an accepted alternative for radical cystectomy[3]. The recurrence rate for muscle-invasive bladder cancer treated with radiotherapy at varying intervals is reported to be 43.6%. (Table 1)[4]. Because the recurrent tumor is of the same histological type as the primary tumor, this is not a second malignancy from previous radiation therapy[5].

In the past, follow-up of patients with bladder cancer is usually 5–10 years[6].There is no definitive consensus on the duration of follow-up for the patients with bladder cancer treated with bladder-conserving methods[7]. Recently, more and more urologists are favoring the idea of lifelong follow-up. The American Urological Association recommends post-treatment surveillance every 3–6 months, with cystoscopies for the initial 3 years and at least yearly later on.

We suggest that all patients with muscle-invasive bladder cancer who have undergone bladder-sparing treatment should have urologic follow-up throughout their life. They should all be counseled about the importance and need of prolonged surveillance, and the risk of delayed local recurrence.

## ACKNOWLEDGMENT

Financial support was received from “CURED 2007” and Mr. Vincent A. Rodriguez.
